# The Theory of Planned Behavior and the Social Identity Approach: A New Look at Group Processes and Social Norms in the Context of Student Binge Drinking

**DOI:** 10.5964/ejop.v16i3.1900

**Published:** 2020-08-31

**Authors:** Loren Willis, Eunro Lee, Katherine J. Reynolds, Kathleen A. Klik

**Affiliations:** a Research School of Psychology, Australian National University, Canberra, Australia; b School of Health and Biomedical Sciences, Royal Melbourne Institute of Technology, Melbourne, Australia; c Research School of Psychology, Australian National University, Canberra, Australia; d Department of Psychology, University of South Carolina, Sumter, SC, USA; ZPID – Leibniz Institute for Psychology Information, Trier, Germany

**Keywords:** theory of planned behavior, social identity approach, binge drinking, social norms, group membership

## Abstract

The current study investigates the theory of planned behavior with important additional predictors from the social identity approach. The study explores whether social identity might function as a driver of the theory of planned behavior and help explain how abstract group processes might impact student binge drinking behavior. Adopting a controlled statistical analysis, the hypothesized model expands the theory of planned behavior’s current conceptualization of group norms and considers how the behavioral content of a specific group, with group identification, impacts binge drinking behavior (N = 551 university students). A path analysis that simultaneously mapped all the hypothesized relationships supported a reconceptualization of social identity as a predictor within the theory of planned behavior. The interaction between group identification and the importance of drinking to the group’s identity significantly predicted an individual’s attitudes towards binge drinking and perceived social binge drinking norms (subjective, descriptive and injunctive), which in turn predicted intentions to binge drink. Intentions to binge drink predicted self-reported binge drinking behavior two weeks later, above and beyond relevant covariates. The implications of these findings are discussed, with recommendations for future research.

Despite considerable effort, researchers are still striving to understand binge drinking behavior, as alcohol remains a leading contributor to burden of disease. Harmful alcohol use is responsible for 1 in 20 deaths worldwide, which is an estimated 3 million people a year (World Health Organization, 2018). The population that reports the highest rates of single occasion risky drinking and the highest levels of alcohol-related accidents or injuries is disproportionately young people (aged 18-24) and students (Australian Institute of Health and Welfare, 2016; National Health and Medical Research Council, 2009). In order to decrease harmful binge drinking behavior, particularly amongst student populations, it is crucial to understand what influences individuals to drink.

Many researchers have utilized the theory of planned behavior (TPB; Ajzen, 1985, 1991) to understand what leads individuals to participate in drinking. The TPB suggests that an individual’s intentions, defined as one’s readiness to perform a behavior, are the best predictors of whether an individual will undertake a given behavior (Godin & Kok, 1996). In turn, one’s intentions are thought to be predicted by their attitudes (one’s positive or negative evaluation of performing the behavior), subjective norms (perception of whether important others think they should perform the behavior or not) and perceived behavioral control (PBC; one’s perceived sense of control over performing the behavior). Underlying attitudes, subjective norms and PBC are behavioral, normative and control beliefs respectively. The TPB has proven to be a successful prediction tool, with a recent meta-analysis concluding that the TPB has a medium to large effect size in predicting intentions to consume alcohol and alcohol consumption (Cooke, Dahdah, Norman, & French, 2016).

The TPB also recognizes a range of possible background variables that might explain why individuals hold certain beliefs, which inform their attitudes, norm perceptions and PBC in a given context. Depending on the target behavior, different salient background variables, such as education, media exposure and neighborhood quality, might indirectly impact intentions and behavior or affect the relationship between attitudes, subjective norms and PBC with intentions (Fishbein & Ajzen, 2010). Many past researchers have explored how demographics, such as age and gender, function as a TPB background variable to predict drinking (Armitage, Norman, & Conner, 2002). Similarly, numerous studies have explored how individual differences and personality traits, like sensation seeking, act as background variables in health behavior prediction (Turchik & Gidycz, 2012). However, psychosocial background variables in the TPB, such as one’s psychological group membership, are not well documented. Based on past binge drinking research, it is evident that group identification can have a considerable impact on student attitudes, norm perceptions and drinking behavior (Hagger, Anderson, Kyriakaki, & Darkings, 2007; Johnston & White, 2003). Therefore, it might be wise to consider social identity processes as a background factor in the TPB when predicting student binge drinking.

In the current study, a possible extension of the TPB is proposed with respect to further emphasis on the social identity approach and group norms. A central idea is that people belong to a number of different groups which can shape their self-concept. Shifts in self-identities may impact on attitudes and inform which ‘others’ and standards will be proponent in shaping one’s own behavior. As a result, when explaining behaviors such as binge drinking, it may be helpful to look at group-level processes in more detail. More specifically, it might be important to consider people’s group memberships, whether drinking is central to what it means to be a group member and the level of connection the individual has to the group (social identification). As is explained in more detail below, these social identity predictors have rarely been explored in relation to the TPB.

The current research proposes that in some instances, social identity might function as a driving background variable in the TPB and therefore make a significant contribution to explaining behavior. Being oriented to a situation as a group member may help explain variability in attitudes, norms and PBC. These key TPB constructs, in part, could be viewed as outcomes of different self-concepts becoming salient in any given context. Furthermore, if a group identity is made salient, specific group norms might have a direct impact on intentions and become highly relevant to behavioral decisions.

## The TPB and the Role of Group Norms

The TPB has received extensive support, but there is still scope to improve the current model’s predictive power. One area of improvement that warrants attention is the model’s conceptualization of normative influence. The only direct variable in the original TPB that considers social pressure is subjective norms. Yet, subjective norms fail to significantly predict health intentions across a number of studies (Godin & Kok, 1996; Johnston & White, 2003; Terry, Hogg, & White, 1999). Armitage and Conner's (2001) meta-analysis concluded that the relationship between subjective norms and intention is consistently the weakest relationship within the model. However, instead of concluding that social norms are not relevant to intentions, perhaps we need to expand our understanding of social norms within the TPB.

While the concept of subjective norms intends to consider all kinds of important social expectations, the way it has been traditionally measured, both directly and indirectly, might limit this process. Participants are usually asked whether most people who are important to them approve or disapprove of the target behavior. Particularly in the context of drinking, this additive approach raises the issue that in an individual’s social network some would typically approve of drinking, such as peers, whereas others would typically disapprove of drinking, such as parents. Similarly, as items do not specify who participants should think about, some participants might answer questions with individuals in mind, such as their grandmother, and others with larger groups, such as their extended family, making the results hard to interpret.

To understand more about which ‘others’ participants are thinking about when perceiving subjective norms, Ajzen and Fishbein (1980) proposed that in pilot studies, participants should identify which individuals and groups would support or not support their participation in a target behavior. Although eliciting these normative beliefs can help us understand who participants have in mind when completing direct subjective norm measures, it is common for participants to also list irrelevant people or groups that do not play a significant role in their decision making. For example, French and Cooke (2012) found that out of 190 participants, 23% listed ‘role models’ and 12% listed non-drinkers, as people who would disapprove of drinking. However, when analyzed further, these groups appeared to have no significant effect on subjective norms or intentions to drink alcohol.

Additionally, as participants are asked questions about ‘important people’, this can implicitly lead to a focus on specific individuals, such as a sister or brother (Donald & Cooper, 2001). Other groups that could be highly important to the individual and have an impact on their decisions, such as their nationality or their university, are potentially overlooked. Abstract groups might be neglected, as they are harder to consciously recognize as influencers and they are not as salient or readily available as important individuals (Donald & Cooper, 2001). Unless participants are directly asked to think about a specific group when completing normative measures, it is unlikely that we will be able to understand how large groups influence intentions and behavior. To understand more about social influence, it might be necessary to narrow the scope for participants and explicitly mention the group norms we are interested in exploring.

A common extension of the TPB, which has been recommended by Ajzen and Fishbein (2005), is to expand the definition of social norms within the model to include both group specific descriptive norms and injunctive norms. The descriptive norm is an individual’s perception of behavior that is typical for most people to do within the defined group (Cialdini, Reno, & Kallgren, 1990). The injunctive norm is an individual’s perception of what is morally approved, or disapproved, conduct for the defined group (Cialdini et al., 1990). Using the descriptive and injunctive norm, we can be group specific about which norms we are interested in exploring, such as what “the typical students at university X” would do. Not only does this make the measurement of social influence easier to interpret, it is also useful in informing social marketing campaigns, that communicate with widespread messaging about largely relevant groups.

Indeed, the addition of both descriptive and injunctive norms as direct predictors of intentions often has increased the predictive power of the TPB (Rivis & Sheeran, 2003), significantly explaining variance in intentions even after subjective norms have been taken into account (Park, Klein, Smith, & Martell, 2009). For example, a meta-analysis conducted by Rivis and Sheeran (2003) concluded that there was a medium to strong correlation between descriptive norms and intentions, with descriptive norms contributing five per cent of explained variance in intentions beyond that of the original TPB variables. Knowing not only what group members approve of, but also whether members act in line with these moral values, can have a large impact on group member intention. However, this extended model still falls short of capturing the group-based processes that affect the perception and impact of social norms (Reynolds, Subašić, & Tindall, 2015).

One influential approach called the social identity approach (SIA) might offer some further insights. The SIA incorporates both social identity theory (Tajfel & Turner, 1979) and self-categorization theory (Turner, Hogg, Oakes, Reicher, & Wetherell, 1987), into one comprehensive approach. It provides a detailed analysis of how social processes such as norms can influence drinking and other behaviors. Through the idea that a person’s self-concept is variable depending on the context a person encounters (being at home or at work, thinking of themselves as a woman or Australian) their attitudes, perceptions and motivations will also vary. This approach may provide a deeper understanding of how group norms and group processes function in the TPB model.

## The Social Identity Approach

The SIA argues that people’s behaviors are influenced by how much they identify as a member of a group. A social identity refers to a person’s knowledge that they belong to a group and that group membership has some emotional and valued significance (Haslam, 2004). It is argued that individuals can identify with multiple groups and as such, have multiple interdependent categorization of the self. As people come to identify and view the group as psychologically meaningful, they internalize the norms, values, and beliefs that define the group, which will help to shape their self-concept (Turner et al., 1987).

Once the group characteristics have been internalized, the group members will have an intrinsic motivation to act in line with the group norms. The group norms impose an internal sanction on the individual to uphold the prototype of the group, which become self-defining and self-reinforcing, as the individual tries to conform to their own self-concept (Hogg & Reid, 2006). Therefore, a group such as one's university is not just an external organization but can be intrinsically tied to an individual’s identity and can have an impact on his or her behavior (Ashforth & Mael, 1989). Thus, it would be advantageous to consider the SIA in conjunction with the TPB to gain a greater understanding of how psychological group processes can impact behavior (Cooke, Sniehotta, & Schüz, 2007; Reynolds et al., 2015).

## The TPB and Social Identity Processes

Building on these ideas, there is a growing body of research that has incorporated group identification with a specific reference group in the TPB model. In the context of the TPB, most studies have analyzed social identification as a moderator of the relationship between group norms and intentions (Johnston & White, 2003; Terry & Hogg, 1996; Terry et al., 1999). For example, Terry and Hogg (1996) found that the perceived norms of a salient reference group influenced intentions to engage in health behaviors, but only for individuals who identified strongly with the in-group. Similarly, Johnston and White (2003) found that beyond the variance explained by attitudes, subjective norms and self-efficacy, group norms moderated by group identification, significantly predicted binge drinking intentions. The group norm was only important for individuals who identified strongly with the student group. These are important developments that reveal psychological connection to the group of interest can help explain drinking and other health behaviors.

Although social identification has been shown to moderate the relationship between perceived norms and behaviour, some researchers have found little support for this relationship. For example, Norman, Clark, and Walker (2005) found that although the addition of descriptive norms did increase the amount of variance explained for aggressive behavior, “little evidence was found to support the moderating role of group identification on TPB-intention relations” (p. 1008). Similarly, Louis, Davies, Smith, and Terry (2007) in a longitudinal study found that student group identification and the perceived healthy eating norm interaction was non-significant in predicting three different measures of healthy eating behavior. Therefore, it may be timely to consider other functions of social identity when it is explored in the context of the TPB.

Perhaps following Fishbein and Ajzen’s (1975) conceptualization that certain background variables operate in the TPB might help us understand more about the role of social identity beyond acting as a moderator in the norm- intention relationship. Ajzen (2011) stated that the TPB variables, particularly attitudes and subjective norms, would be affected by context and vary as a function of the specific population. If the context is conceptualized as the social context and the specific population is thought to be the reference group of interest, then it is possible that social identification might directly influence the variables within the TPB. A salient social identity could make accessible certain beliefs that provide the foundation for norm perceptions, attitudes and PBC.

As stated by Hogg and Smith (2007) “self-categorization transforms self-conception to match the identity described by the category, and transforms one’s perceptions, attitudes, feelings and conduct to conform to the category prototype” (p. 96). In other words, when individuals identify with a salient group, a depersonalization process occurs where the groups norms, values and beliefs, become their own. If a group has positive attitudes towards drinking, it is likely that highly identifying group members will exert aligning attitudes to reflect their valued group membership. If drinking is not central or particularly relevant to the prototype of the group, members attitudes towards drinking will vary based on individual or other group evaluations. Similarly, if many in-group members engage in drinking, this can act as a trusted heuristic and a persuasive cue to drink, affecting individual PBC (Hogg & Smith, 2007). However, if individuals don’t identify with the salient group, they are more likely to rely on personal beliefs, resulting in higher PBC. It is through our understanding of the SIA that we can begin to understand how group processes might impact the TPB variables.

A study by Hagger, Anderson, Kyriakaki, and Darkings (2007) was also interested in the impact of social identity on TPB constructs and, in turn, intentions and behavior. Their results indicated that social identity positively influenced attitudes, subjective norms and PBC for binge drinking, highlighting that it could be a predictor driving the behavioral model. However, social identity did not significantly predict attitudes, PBC or subjective norms in relation to exercise and dieting behavior. A potential reason for the mixed results is that Hagger et al. (2007) incorporated less widely used measures of social identity, characterizing it more as an individual quality related to the importance of popularity and social connections. Moreover, Hagger et al. (2007) explain that exercise and dieting were not seen as social behaviors, and therefore social identity was less likely to have an impact. However, it is possible that exercise and dieting where not important to the group’s identity, and therefore one’s identification with the group did not predict engagement in those behaviors. Whereas binge drinking could have been central to the group’s identity. This interaction between identification and how important a behavior is to the group has not been explored in the context of the TPB.

Past research has predominantly focused on measuring a form of group identification, such as “how much do you feel you identify with (insert group)?” (Terry & Hogg, 1996). However, just knowing whether someone identifies with the group is not enough. We need to understand group identification in the context of what it means to be a part of the group. It is important to understand what behaviors and values characterize the group and are central to the group’s identity, as they form the group content and differentiate it from out-groups (Hogg & Reid, 2006). It is the characteristics that define the group that will shape a group member’s behavior when the group is salient and there is a high level of psychological connection with the group.

Along these lines, Livingstone and McCafferty (2015) demonstrated in an experiment that personal attitudes and group identification moderated whether norm information impacted intentions, but only when the behavior in question was central to the group’s identity (drinking alcohol as opposed to drinking coffee). When norm information was supplied about a behavior that was not group defining, one’s level of identification with the group did not impact intentions to behave. Similarly, Livingstone, Young, and Manstead (2011) found that when they tried to manipulate the descriptive norm to decrease drinking behavior, if binge drinking was perceived as central to the student identity, one had positive attitudes towards drinking and the individual highly identified, a backlash effect occurred, as the group felt their identity was under threat. Therefore, the importance of the behaviour to the group’s identity and meaning (*its’ raison d'être*) needs to be taking into consideration in conjunction with identification levels when predicting student drinking.

## The Present Study

By exploring group processes and social norms from a SIA perspective, we can begin to understand how the social context shapes binge drinking behaviour in the context of the TPB. There are three major gaps in the literature that the current research seeks to address. First, in addition to the subjective norm variable, group specific injunctive and descriptive norms will be added to the model to understand how larger social group norms directly impact intentions to binge drink. Second, instead of exploring social identification as a moderator of the norm – intention relationship, it is proposed that social identity processes act as a background variable within the TPB. Third, instead of just focusing on whether one identifies with a group in order to predict behavior, we will explore how the perceived importance of the behavior relative to what it means to be a group member will impact one’s decision to engage in binge drinking.

The current project also adopts more controlled statistical analyses to understand the TPB. The majority of studies in the TPB drinking literature used multiple hierarchical regressions for analysis (for examples see Barratt & Cooke, 2018; Hasking & Schofield, 2015; Johnston & White, 2003; Norman, Armitage, & Quigley, 2007). A limitation of multiple regression is that it can only have one dependent variable, meaning that the predictors of intention and the predictors of behavior are not entered simultaneously. However, the current study utilizes path analysis within the program Mplus (Version 8.3), which can model multiple dependent variables with simultaneous estimation and account for both direct and indirect effects for a more sophisticated analysis (Jeon, 2015).

Although binge drinking has been a popular focus of past literature, the term ‘binge drinking’ is not well defined and lacks consistency. In the current study, binge drinking is defined as consuming seven or more standard alcoholic drinks on a single occasion (Cooke et al., 2007; Courtney & Polich, 2009; Palmer, Fryer, & Kalafatelis, 2009). Research has shown that after seven or more drinks have been consumed on a single occasion, individuals are at a greater risk of suffering from alcohol related problems and injuries (Fillmore & Jude, 2011). Furthermore, most research in the area has been conducted in the USA, with most researchers using five standard drinks as the binge drinking definition (Gabbiadini, Cristini, Scacchi, & Monaci, 2017; Neighbors, Lee, Lewis, Fossos, & Larimer, 2007). As a standard drink in the USA corresponds to 14g of alcohol, whereas a standard drink in Australia is 10g of alcohol, five standard drinks in the USA is the equivalent to seven standard drinks in Australia (Kerr, Patterson, Koenen, & Greenfield, 2009). Therefore, the current study’s definition of binge drinking is comparable to most of the past literature within the field.^i^

In line with the TPB, it is hypothesized that PBC (H1a) and intentions (H1b) will directly predict drinking behavior. As PBC decreases and binge drinking intentions increase, binge drinking behavior will increase after all covariates are considered. Furthermore, attitudes towards drinking (H2a), subjective norms (H2b) and PBC (H2c) will significantly predict binge drinking intentions. As positive attitudes towards binge drinking increase, subjective drinking norms increase and PBC decreases, one’s intentions to engage in binge drinking will increase.

In addition to the traditional TPB model, it is hypothesized that as group specific descriptive norms increase (H3a) and group specific injunctive norms increase (H3b), binge drinking intentions will also increase. It is further predicted that the interaction between group identification and the importance of drinking to group identity will significantly predict drinking attitudes (H4a), subjective norms (H4b), descriptive norm (H4c), injunctive norm (H4d) and PBC (H4e). The importance of drinking to group identity will be more strongly related to drinking attitudes, drinking norms (subjective, descriptive, and injunctive) and PBC for high group identifiers than for low group identifiers.

## Method

### Pilot Study

An online study was conducted prior to the main study to confirm whether the traditional method of gathering normative beliefs lead participants to neglect larger groups. The pilot consisted of 39 university students; 25 females (64.1%), *M*_Age_ = 21.1 years, *M*_Time spent at university_ = 5.8 semesters. Participants were asked open-ended questions about their drinking behavior, such as which individuals or groups would approve or disapprove of their drinking.^ii^ Two markers reviewed and categorized the responses.

When listing people or groups who approved of binge drinking behavior, only six participants (15%) mentioned a wider social group (such as their residential college or their gender). Similarly, only three participants (8%) mentioned a wider social group (such as health organizations or religious groups) who disapproved of drinking. The remaining participants listed people who were specific to them, such as their parents, older sibling or significant other. It appears that if researchers want to explore the influence of social groups, which might be more relevant when designing large-scale interventions, the group may needs to be explicitly named when measuring social norms in the TPB.

### Main Study

#### Participants

In an Australian University with 25,000 enrolments, a total of 551 students participated in the study. Of the participants, 281 (51%) were living at university residencies (eight different residencies in total) and 270 (49%) had other housing types, such as the family home or rented share houses. The mean length of time spent enrolled at the university was 4.18 semesters (*SD* = 2.75). The sample consisted of 158 (28.7%) males, 325 (59%) females, and 6 (1.1%) people who identified their gender as ‘other’, with the mean age of participants being 20.5 years (*SD* = 2.67, range 18 - 42). Of the 551 people who completed the initial survey, 274 (50%) completed the follow-up behavioral survey after two weeks. Of those respondents, 90% could be linked to their initial survey response via research codes, which resulted in 246 (45%) participants with data from both the initial survey and the follow-up. Following missing value analysis and attrition analysis, measures for participants who did not respond to the follow-up were imputed using multiple imputation in Mplus (Version 8.3), as is recommended for statistical rigor over listwise deletion (Asendorpf, Van De Schoot, Denissen, & Hutteman, 2014; Graham, 2009; Newman, 2014).

#### Procedures

Participants were recruited through an internal research database, on the university’s social media pages and in person. After reading a participant information sheet, all participants completed an online or paper-based questionnaire. Following a two-week period, participants were sent an email asking about their drinking behavior since completing the initial survey. A research code was used to link their responses between the initial survey and the follow-up to maintain anonymity.

At the start of the questionnaire a visual representation of Australian standard drinks was presented. The surveys were customized to reflect a relevant reference group for the participant. If the participant was a member of a university residency, their reference group became ‘other (college) students’, with their residential college name inserted in each relevant item. If a participant was not a remember of a residency, their reference group became ‘other (university) students’, with the university’s name inserted in each relevant item. Measures were constructed in line with Ajzen’s (2006) instructions, using the target, action, context, time (TACT) method (Conner & Sparks, 2005; Francis et al., 2004), with “7 or more standard alcoholic drinks on a single occasion over the next 2 weeks” as the stem of all questions. Unless otherwise stated, responses were recorded on a 7-point scale ranging from 1 (*strongly disagree*) to 7 (*strongly agree*).

#### Measures

##### Control variables

The covariates of binge drinking in the current study include the participants’ age, gender, how many semesters they have been enrolled at the university and the participants’ past binge drinking behavior^iii^. Past research has indicated that these control variables are associated with student drinking behavior (Barratt & Cooke, 2018; Neighbors et al., 2010; Reed, Lange, Ketchie, & Clapp, 2007; Rimal & Real, 2005). Past drinking behavior was measured using the Daily Drinking Questionnaire (DDQ; Collins, Parks, & Marlatt, 1985; Dimeff, Baer, Kivlahan, & Marlatt, 1999). The participants were asked to report the number of standard drinks they consumed on every day of their heaviest drinking week within the past month^iv^. If the participant indicated that they had drunk seven or more standard drinks on at least one occasion within the week, then their past behavior was coded as ‘1’ for binge. If there was no occasion where the participant drunk seven or more standard drinks, they were coded as ‘0’ for no binge. The DDQ has been used extensively in past research on student drinking and has good test-retest reliability and good convergent validity (Marlatt et al., 1998; Neighbors, Lewis, Bergstrom, & Larimer, 2006).

##### Group identification

Group identification was measured on a three-item scale of identity centrality (α = .89), adapted from Leach et al. (2008). Items included “The fact that I am a student at [insert group] is an important part of my identity” and “Being a student at [insert group] is an important part of how I see myself”^v^.

##### Importance of drinking to the group

The importance of drinking to the in-group was measured on a 7-item scale (α = .88) adapted from Livingstone et al. (2011). Items included “If I had to pick one thing that defines being a student at [insert group], it would be drinking alcohol” and “Drinking is central to the image of being a student at [insert group]”.

##### Attitude

Attitude was measured using a 7-point semantic differential scale with seven differential adjectives (α = .92). Participants responded to the statement “Drinking more than 7 standard alcoholic drinks on a single occasion over the next two weeks would be…” anchored with semantics such as Unenjoyable-Enjoyable, Good-Bad, Foolish-Wise. Higher scores indicate pro-drinking attitudes.

##### Perceived behavioral control

PBC was measured with two items (α = .60). The items measured were “I feel in complete control of whether I drink more than 7 standard alcoholic drinks in a single occasion over the next two weeks” and “How much personal control do you have over whether you drink 7 or more standard alcoholic drinks on a single occasion over the next two weeks?” from *No control at all* (1) *– Complete control* (7).

##### Subjective norms

Subjective norms for pro-drinking were measured using three items (α = .83). All items referred to ‘most people who are important to me’, with items such as “Most people who are important to me expect me to drink 7 or more standard alcoholic drinks on a single occasion over the next two weeks” and “Most people who are important to me think I *should not* (1) *- should* (7) drink 7 or more standard alcoholic drinks on a single occasion over the next two weeks”.

##### Injunctive norm

Injunctive norms for pro-drinking were measured using two items (α = .69) on a 7-point scale. The items included “Most student at [insert group] think I *should not* (1) *- should* (7) drink 7 or more standard alcoholic drinks on a single occasion over the next two weeks” and “If I drank 7 or more standard alcoholic drinks on a single occasion over the next two weeks, most students at [insert group] would *approve* (7) *- disapprove* (1)”.

##### Descriptive norm

The Drinking Norms Rating Form (DNRF; Baer, Stacy, & Larimer, 1991) was used to measure perceptions of the drinking descriptive norm. Participants were asked to estimate the number of standard drinks consumed on each day of a typical week within the last month by a typical member of their group. If an estimation on any day of the week indicated the norm was seven or more standard drinks, the descriptive norm was coded as ‘1’ for binge. If there were no days were seven standard drinks was exceeded, the norm was coded as ‘0’ for no binge.

##### Intentions

Intentions were measured on a three-item scale of generalized intentions to drink (α = .97). Participants responded to the items “*I intend/I want/I expect* to drink more than 7 standard alcoholic drinks on a single occasion over the next two weeks”. Descriptive statistic results showed that intentions were over-dispersed and polarized, with people either having extremely low or extremely high intentions to drink. Therefore, based on the distribution of the data, a binary variable of intentions was created. If participants’ intentions fell on or below the midpoint of 4, they were coded as ‘0’ for low intentions and if their intentions fell above the midpoint of 4, they were coded as ‘1’ for high intentions.

##### Behavior

Binge drinking behavior was measured during a follow-up, two weeks after the initial questionnaire. Participants were asked to recall their drinking behavior since the initial survey, recording the information using the DDQ (Collins et al., 1985). If the participant indicated that they had drunk more than seven standard drinks on one occasion within the two weeks, they were coded as ‘1’ for binge. If there was no occasion where the participant drunk more than seven standard drinks, they were coded as ‘0’ for no binge, to create an overall binary binge drinking variable.^vi^

### Analysis Overview

Descriptive statistics analysis was conducted in SPSS (Version 22). The nested nature of the data was considered, as students from the same residential college could be more correlated compared to students from other residential colleges, or non-residents. Therefore, Mplus (Version 8.3) was used to calculate interclass correlation coefficients (ICC) and design effects to determine whether the assumption of independent observations had been violated (Hox, 2010). As all the ICCs were greater than zero (range .04 - .21) and the design effects were greater than two (range 3.22 - 12.25) residency was used as a cluster variable with TYPE = COMPLEX in Mplus (Version 8.3) for the imputation and the main analysis (Muthén & Satorra, 1995).

Missing values analysis was conducted in SPSS using EM with 50 iterations. Little’s missing completely at random (MCAR) test was non-significant (χ^2^ = 159.79, *df* = 141, *p* = .133) indicating that missingness was completely at random. Therefore, as recommended with MCAR and MAR data, multiple imputation with 40 iterations in Mplus was used to impute the missing data (Newman, 2014) and Mplus combined the results into a single set of estimates and standard errors.

All study variables had a significant Shapiro-Wilk statistic (all *p* < .001), with visual inspection of histograms and Q-Q plots suggesting that the data was non-normal, as many of the variables were skewed. Therefore, the maximum likelihood estimator with robust standard errors (MLR) in Mplus was used to account for the non-normality violations. Construct validity of the measures was examined with the measurement models in Mplus, with modification indices applied. CFA confirmed that all items adequately loaded onto their respective latent factor, ranging from .56 (good) to .98 (excellent), except for item 4 in the ‘importance of drinking to the group’ measure, which had a poor loading of .36 (Comrey & Lee, 1992) and was subsequently dropped from the main analysis.

Logistic regression path analysis was conducted to model all the predictors of binge drinking behavior (refer to Table 4). The TPB explanatory variables, social norms, group identification and the importance of drinking to the group were included in the final model, while the demographic covariates and past drinking behavior were taken into account. The main effects of group identification and the importance of drinking to the group (along with the interaction terms) were entered into the model because the significance of the multiplicative term cannot be tested without controlling for the component main effects (Cohen & Cohen, 1983). The main effects of group identification and the importance of drinking to the group on TPB variables will only be interpreted if the interaction effect is non-significant. Mediators were PBC, attitudes, subjective norms, injunctive group norms and descriptive group norms, which in turn were hypothesized to impact binge drinking intentions. It was also predicted that the final outcome variable, binge drinking, would be impacted by PBC and the proximal mediator drinking intentions. Odds ratios were reported for the relationships with binary variables, by exponentiating the estimated coefficients. As the odds ratio terms are multiplicative, positive effects are odds ratios that are greater than one and negative effects are between zero and one. McFadden’s adjusted *R*^2^ was utilized to determine model fit, with values above .2 representing excellent model fit (McFadden, 1977).

## Results

### Descriptive Statistics

Means, standard deviations, skew, kurtosis and alpha levels are presented in Table 1.

**Table 1:** Descriptive Statistics of the Scale Scores

Variable	*M*	*SD*	Skew	Kurtosis	α
Injunctive norm	4.2	1.33	-.22	-.06	.69^a^
Subjective norm	3.45	1.56	.11	-.67	.83
Perceived behavioral control	5.87	1.33	-.12	.82	.60^a^
Attitudes	3.70	1.43	-.37	-.51	.92
Social identity	4.48	1.6	-.42	-.65	.89
Importance of drinking	3.42	1.15	.34	-.12	.86

^a^The injunctive norms and PBC measures showed a Cronbach α smaller than .7, the recommended reliability level. However, the measures only had two indicator items respectively, so the α is simply a correlation of the two items. Considering the sensitivity of the α to measures with a small number of items, the measures were retained in the model.

In the last month, 192 students (35%) recorded that they had participated in binge drinking within a typical week and 302 (55%) participated in binge drinking during a heavy week of drinking. Overall, 250 (45.5%) participants were coded as intending to binge drink during the two weeks following the questionnaire. Most students perceived that the descriptive norm involved binge drinking, as 308 (58.4%) students indicated that the typical student in their group would binge drink at least once in a typical week. Of the 245 participants who completed the follow-up, 82 (33.5%) self-reported binge drinking behavior within the two-week period. The number of drinks on a given day ranged from 0 to 25 drinks. Correlations among the study variables are presented in Table 2.

**Table 2:** Correlations Among Variables

Variable	Correlation													
	1	2	3	4	5	6	7	8	9	10	11	12	13	14
1. Behavior	-													
2. Intentions	.54**	-												
3. PBC	-.35**	-.39**	-											
4. Attitudes	.47**	.60**	-.34**	-										
5. Subjective norm	.40**	.57**	-.36**	.64**	-									
6. Injunctive norm	.26**	.34**	-.21**	.39**	.49**	-								
7. Descriptive norm	.28**	.36**	-.23**	.31**	.35**	.38**	-							
8. Importance	.19**	.21**	-.25**	.12**	.27**	.38**	.23**	-						
9. Identification	.06	.07	-.13**	.05	.07	.07	.10*	.10*	-					
10. Past behavior	.51**	.62**	-.39**	.57**	.54**	.32**	.45**	.18**	.09*	-				
11. Age	-.09	-.17**	.09*	-.15**	-.15**	-.16**	-.10*	-.08	.01	-.15**	-			
12. Time at university	.06	-.01	.08	.03	.05	.05	.10*	.02	.06	.05	.44**	-		
13. Female	-.05	-.09	.05	-.12*	-.09*	.06	.09*	.08	.06	-.09	-.10*	-.02	-	
14. Other gender	-.02	-.07	.07	-.08	-.11*	-.03	-.06	.01	-.01	-.05	-.01	-.01	-.16**	-

*Note*. Behavior: 0 = no binge, 1 = binge. Intentions: 0 = no intentions to binge, 1 = intentions to binge. Descriptive norm: 0 = no binge, 1 = binge. Past behavior: 0 = no binge, 1 = binge. Female: 0 = male, 1 = female. Other Gender: 0 = male, 1 = other gender identifiers.

*p < .05. **p < .01.

### Attrition Analysis

Attrition analysis was conducted in SPSS (Version 22) before the missing data imputation, as seen in Table 3. Due to the unequal group sizes between the initial and follow-up participants, regression analysis was conducted to see whether people who completed the follow-up behavior measure were significantly different on variables of interest compared to the people who dropped out. The results showed that people who participated in the follow-up were significantly more likely to report lower drinking behavior in the past for both a typical week (*M* = 6.87 compared to 9.74) and a heavy drinking week (*M* = 14.2 compared to 17.3), lower perceptions of subjective norms for drinking (*M* = 3.27 compared to 3.63), higher PBC (*M* = 6.07 compared to 5.67), more negative attitudes towards drinking (*M* = 3.49 compared to 3.9) and were more likely to be female. All other variables were not significantly different between the two groups. Multiple imputation was used so that a more representative sample would be analyzed, but the results should be interpreted with caution considering the attrition results.

**Table 3:** Attrition Analysis Results: Regression Coefficients

Variable	*Β*	*SE*
Past behavior: Heavy	-0.09*	1.54
PBC	0.15***	0.12
Intentions	-0.05	0.20
Attitudes	0.04***	0.13
Descriptive norm	0.03	0.81
Group identification	0.04	0.10
Injunctive norm	-0.00	0.12
Importance of drinking	0.01	0.10
Subjective norm	-0.11*	0.14

*Note.* Dropout group = 0. Group with follow-up data = 1. PBC = Perceived behavioral control. *SE* = Standard error.

*p < .05. ***p < .001.

### Main Logistic Regression: Path Analysis

**Figure 1:**
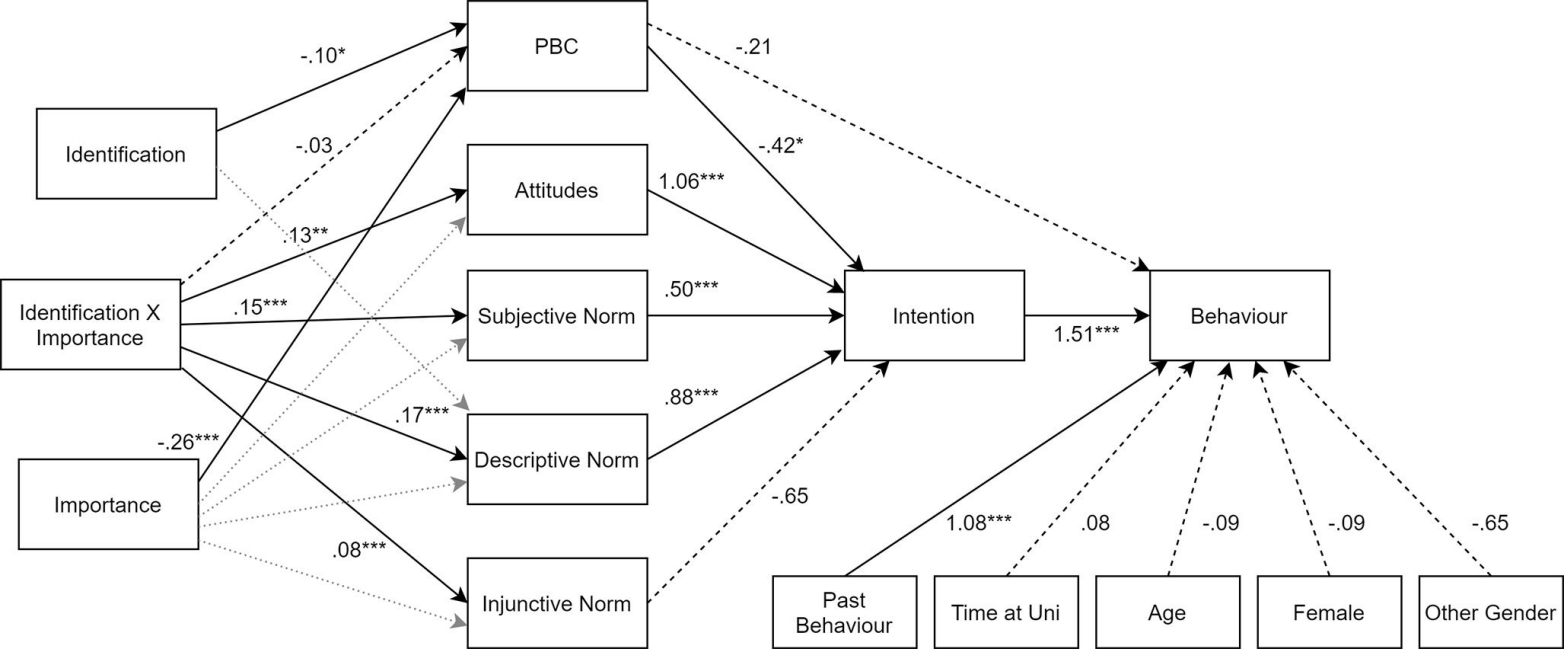
Logistic regression path analysis of the hypothesized model (Model 4). *Note*. Behavior: 0 = no binge, 1 = binge. Intentions: 0 = no intentions to binge, 1 = intentions to binge. Descriptive norm: 0 = no binge, 1 = binge. Past behavior: 0 = no binge, 1 = binge. Female: 0 = male, 1 = female. Other Gender: 0 = male, 1 = other gender identifiers. Coefficients are unstandardized. Dashed lines represent non-significant pathways, while dotted grey lines depict the main effect paths that were significant but overridden by the significant interaction effects. Non-significant main effect paths, error terms, and correlations are excluded from the diagram for visual simplicity. *p < .05. **p < .01. ***p < .001.

Logistic regression path analysis with observed variables was utilized to test the hypotheses, with a hierarchal model presented in Table 4. The final hypothesized model results are discussed below and presented in Figure 1. The global model fit significantly improved when all the hypothesized predictors were included compared to the intercept model, $\chi^{2}$(*df* = 27, *N* = 551) = 6516.68, *p* < .001, with McFadden’s adjusted *R*^2^ = .58, showing excellent model fit (Veall & Zimmermann, 1996).

Past drinking behavior was the only covariate to significantly predict binge drinking behavior (*b* = 1.08, *p* < .001), as the odds ratio of binge drinking behavior increases by a factor of 2.95 for every unit increase in past drinking behavior. If a participant self-reported binge drinking behavior anytime in the month prior to the survey, they were more likely to report binge drinking in the follow-up. All other demographic variables were nonsignificant in predicting binge drinking behavior. It also appeared that PBC did not directly predict binge drinking behavior (*b* = -0.21, *p* = .087). Contrary to predictions, a decrease in PBC did not predict an increase in drinking behavior (H1a). Intentions positively influenced binge drinking behavior (*b* = 1.51, *p* < .001), as the odds ratio of binge drinking behavior increases by a factor of 4.52 for every unit increase in drinking intentions. As hypothesized, the more an individual intended to binge drink, the more likely they were to participate in binge drinking within the two-week follow-up (H1b).

In support of the original TPB, drinking attitudes, subjective norms and PBC all emerged as significant predictors of binge drinking intentions. Attitudes positively determined intentions (*b* = 1.06, *p* < .001). The odds ratio of binge drinking intentions increases by a factor of 2.89 for every unit increase in positive attitudes towards drinking. As positive attitudes towards binge drinking increased, intentions to engage in binge drinking increased significantly, as was originally hypothesized (H2a). Subjective norms positively determined intentions (*b* = 0.50, *p* < .001), with the odds ratio of binge drinking intentions increasing by a factor of 1.65 for every unit increase in subjective norms. In line with predictions, if an individual perceived that most people who were important to them approved of binge drinking, they were more likely to intend to binge drink themselves (H2b). PBC negatively predicts intentions (*b* = -0.42, *p* = .012), as the odds ratio of binge drinking intentions is 0.66 times as likely for every unit increase in PBC. As feelings of PBC decreases, intentions to binge drink increase, as was hypothesized (H2c).

Group-specific descriptive drinking norms and group-specific injunctive drinking norms were predicted to have a significant relationship with drinking intentions. Perceived descriptive norms positively predicted intentions (*b* = 0.88, *p* < .001), with the odds ratio of binge drinking intentions increasing by a factor of 2.42 for every unit increase in descriptive norms. In accordance with the hypothesis, the more an individual perceived that a typical member of their group would engage in binge drinking behavior in a typical week, the greater their own binge drinking intentions (H3a). Unexpectedly, injunctive norms did not significantly predict intentions (*b* = 0.06, *p* = .52). Hypothesis H3b was not supported, as the approval or disapproval of the specified student group did not appear to impact individual intentions to binge drink.

It was also predicted that the group identification and importance of drinking to the group identity interaction would directly predict attitudes, perceived social norms and PBC. The interaction between identification × importance did significantly predicted attitudes (*b* = 0.132, *p* = .005), subjective norms (*b* = 0.146, *p* < .001), descriptive norms (*b* = 0.168, *p* < .001) and injunctive norms (*b* = 0.077, *p* < .001). As was hypothesized, drinking attitudes (H4a), subjective norms (H4b), descriptive norms (H4c) and injunctive norms (H4d) were all influenced by whether one identified with the relevant group and whether drinking was important to the group’s identity. However, identification × importance did not significantly predict PBC (*b* = -0.027, *p* = .463). Contrary to the hypothesis, there was no moderated relationship between importance of drinking to the group, group identification and PBC (H4e). Yet, the main effects of identification (*b* = -0.096, *p* = .018) and importance of drinking to the group (*b* = -0.255, *p* < .001) were significant negative predictors of PBC. Although they were not predicted effects, it appears that the higher one’s identification with the group, the lower their PBC. Additionally, as one’s perception of the importance of drinking to the group identity increases, their PBC decreases.^vii^

**Table 4:** Coefficients of Logistic Regression Path Analysis: Hierarchical Models to Explain Binge Drinking Behavior and Intentions with TPB and SIA

Predictors	Model 1			Model 2			Model 3			Model 4		
	*b*	*SE*	OR	*b*	*SE*	OR	*b*	*SE*	OR	*b*	*SE*	OR
Behavior predictors												
Past behavior	2.06***	0.29	7.85	1.08**	0.26	2.95	1.08**	0.26	2.95	1.08**	0.26	2.95
Age	-0.11*	0.07	0.9	-0.09	0.07	0.92	-0.09	0.07	0.92	-0.09	0.07	0.92
Time at Uni	0.07*	0.06	1.07	0.08	0.06	1.08	0.08	0.06	1.08	0.08	0.06	1.08
Female	-0.17	0.25	0.84	-0.09	0.28	0.92	-0.09	0.28	0.92	-0.09	0.28	0.92
Other gender	-1.21	1.23	0.3	-0.65	1.08	0.52	-0.65	1.08	0.52	-0.65	1.08	0.52
Intentions				1.51***	0.33	4.52	1.51***	0.33	4.52	1.51***	0.33	4.52
PBC				-0.21	0.12	0.81	-0.21	0.12	0.81	-0.21	0.12	0.81
Intention predictors												
PBC				-0.45**	0.17	0.64	-0.42**	0.17	0.66	-0.42**	0.17	0.66
Attitudes				1.07***	0.12	2.9	1.06***	0.13	2.89	1.06***	0.13	2.89
Sub norm				0.58***	0.11	1.78	0.50***	0.1	1.64	0.50***	0.1	1.64
Des norm							0.88***	0.2	2.42	0.88***	0.2	2.42
Injun norm							0.06	0.1	1.06	0.06	0.1	1.06
PBC predictors												
Identification										-0.10*	0.04	
Importance										-0.26***	0.05	
Iden X Imp										-0.03	0.04	
Attitude predictors												
Identification										0.05	0.06	
Importance										0.14*	0.06	
Iden X Imp										0.32**	0.05	
Subjective norm predictors												
Identification										0.05	0.05	
Importance										0.33***	0.07	
Iden X Imp										0.15***	0.04	
Descriptive norm predictors												
Identification										0.13**	0.05	1.11
Importance										0.42***	0.08	1.48
Iden X Imp										0.17***	0.03	1.19
Injunctive norm predictors												
Identification										0.03	0.05	
Importance										0.39***	0.05	
Iden X Imp										0.08	0.02	
Model information												
Loglikelihood difference (χ^2^)	χ^2^(*df* = 5, *N* = 551)			χ^2^(*df* = 5, *N* = 551)			χ^2^(*df* = 2, *N* = 551)			χ^2^(*df* = 15, *N* = 551)		
	= 60.75, *p* < .001			= 184.71, *p* < .001			= 6.86, *p *< .05			= 137.99,* p *< .001		

*Note. SE =* Standard error. OR = Odds ratio. Sub norm = Subjective norm. Des norm = Descriptive norm. Injun norm = Injunctive norm. Importance = Importance of drinking to the group. Iden X Imp = Interaction between identification and importance of drinking to the group. Behavior: 0 = no binge, 1 = binge. Intentions: 0 = no intentions to binge, 1 = intentions to binge. Descriptive norm: 0 = no binge, 1 = binge. Past behavior: 0 = no binge, 1 = binge. Female: 0 = male, 1 = female. Other gender: 0 = male, 1 = other gender identifiers. Coefficients are unstandardized.

*p < .05. **p < .01. ***p < .001.

### The Main Model Interaction Effects

#### Group Identification Moderating the Impact of Importance of Drinking to the Group on Attitudes, Perceived Behavioral Control and Social Norms

**Figure 2:**
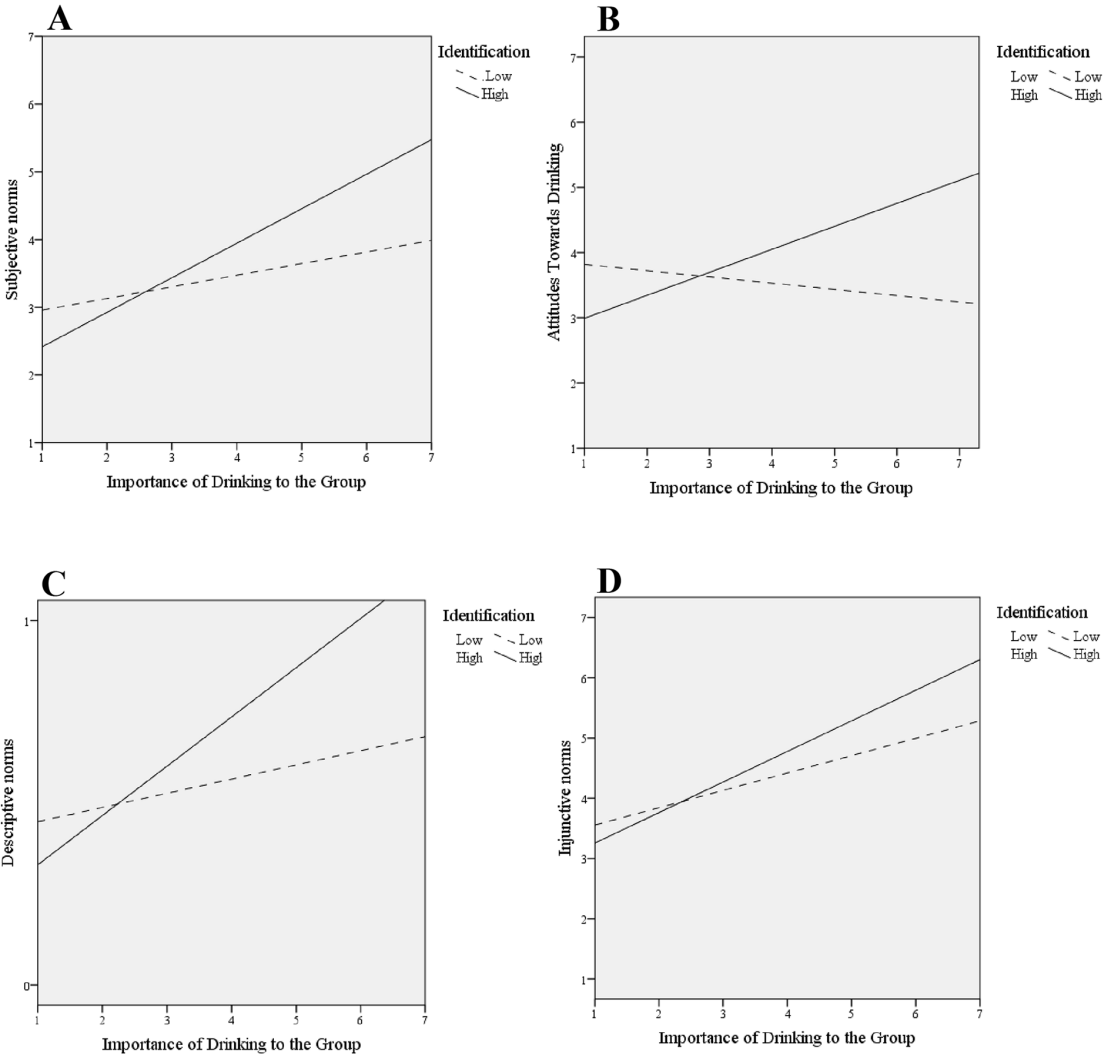
Group identification as a moderator of perceived importance of drinking to the group in explaining (A) subjective norms, (B) attitudes, (C) descriptive norms, and (D) injunctive norms.

A second set of analyses plotted the significant group identification × importance of drinking to the group identity effects, in order to visualize the relationships. The significant interaction effects were explored in SPSS (Version 22; as seen in Figure 2) and simple slopes estimated from parameter estimates are presented, with identification split into low identifiers and high identifiers, and perceived importance of drinking to the group on the x-axis^viii^. However, SPSS (Version 22) does not have a cluster analysis option, therefore the following results include confounding from intraclass correlations within residency groups and should be interpreted with caution.

##### Subjective norms

The positive association between perceived subjective norms and the importance of drinking to the group was stronger for students who highly identified with the group compared to students who had low levels of identification.

##### Attitudes

There was a positive association between attitudes towards drinking and the importance of drinking to the group when students highly identified with the group. However, the opposite was true for low identifiers; there was a negative association between importance of drinking to the group and attitudes when the student had a low level of identification.

##### Descriptive norms

As the perceived importance of drinking to the group increase, so did the perceived descriptive norm. The positive relationship between descriptive norms and the importance of drinking to the group was stronger for students who highly identified with the group compared to students who had low identification levels.

##### Injunctive norms

The positive association between perceived injunctive norms and the importance of drinking to the group was stronger for students who highly identified with the group compared to students who had low identification levels.

## Discussion

The present study sought to explore SIA constructs as background variables within the TPB and to understand how group specific norms predict intentions. The aim was to explore whether group membership (including identification and identity meaning) could be reconceptualized to act as a driver of the TPB when predicting binge drinking in university students. Overall, there appears to be support for exploring social identity as an additional predictor in the TPB, with group processes predicting individual perceptions, including group specific norms, that affect drinking behavior.

With regards to Hypothesis 1b, intentions did significantly predict binge drinking behavior, as was expected and consistent with past research. If a student intended to participate in binge drinking, they were more likely to self-report binge drinking behavior two weeks later. In line with the original TPB, it was also hypothesized that PBC would directly predict behavior (H1a), which was not supported. However, numerous studies have found the relationship between PBC and drinking behavior to be non-significant (Cooke et al., 2007; Hagger et al., 2007; Terry & Hogg, 1996; Terry et al., 1999). A meta-analysis conducted by Cooke et al. (2016) confirmed that the relationship between drinking behavior and PBC was small, negative and non-significant, with many of the reviewed studies showing inconsistent results. Therefore, it appears that the current study’s results are consistent with other TPB models in the context of student binge drinking.

The other hypotheses regarding the original TPB model were supported, as attitudes (H2a), subjective norms (H2b) and PBC (H2c) all predicted drinking intentions. As attitudes towards drinking became more positive and perceptions of pro-drinking subjective norms increased, overall intentions to engage in binge drinking increased, as is consistent with the TPB. Furthermore, the less PBC one felt they had over the decision to binge drink, the more likely that they intended to drink. Although the relationship between PBC and intentions is sometimes theorized to be positive, in relation to drinking behavior, the significant negative relationship is in line with past research (Cooke et al., 2016; Gabbiadini et al., 2017). It is possible that for negatively evaluated and/or traditionally socially undesirable behaviors, greater perceptions of control lead to lower intentions to act (Cooke et al., 2016; Eagly & Chaiken, 1993).

Hypothesis 3 was partly supported. Binge drinking descriptive norms did significantly predict intentions (H3a), but injunctive norms did not (H3b). As is consistent with past research, the more one perceived everyone else in the group was binge drinking, the greater their intentions to engage in binge drinking themselves (Rimal & Real, 2005; Rivis & Sheeran, 2003). Although it was hypothesized that the more one perceived members of their group to approve of drinking, the more one would intend to drink, injunctive norms did not significantly predict intentions. Looking back at past research, when most studies have extended the TPB to include injunctive norms, researchers have used a measure of ‘group norms’, which combines both descriptive and injunctive norm items (for examples see Johnston & White, 2003; Terry & Hogg, 1996; Terry et al., 1999; White, Smith, Terry, Greenslade, & McKimmie, 2009). As descriptive and injunctive norms have been shown to operate differently when affecting drinking intentions and behavior (Cho, 2006), using a group norm measure means that it is impossible to disentangle the normative influence.

Of the research that does specifically measure injunctive group norms and drinking intentions, the results are mixed. Some researchers have concluded that the main effect of injunctive norms on intentions and/or drinking behavior is only significant when the reference group is in close proximity to the individual (Cho, 2006; Park et al., 2009). For example, Reed, Lange, Ketchie, and Clapp (2007) found that injunctive norms predicted drinking behavior when the reference group was friends, but not peers or Greek-letter members. It is possible that the reference group in the current study was too distal for the injunctive norms to influence intentions. Additionally, a common occurrence when injunctive and descriptive norms are entered into a model simultaneously, is that injunctive norms acts as a suppressor variable, resulting in a strong relationship between descriptive norms and intentions, and a non-significant relationship between injunctive norms and intensions (Chawla, Neighbors, Lewis, Lee, & Larimer, 2007; Manning, 2009, 2011; Neighbors et al., 2008). Alternatively, as subjective norms predicted intentions, but injunctive norms did not, it is possible that the approval of important individuals, like one’s best friend and parent, is more relevant than the approval of larger groups when an individual decides to binge drink. More research is needed to clearly understand the relationship (or lack thereof) between injunctive norms and drinking intentions.

The interaction between group identification and the importance of drinking to the group did significantly predicted attitudes (H4a), subjective norms (H4b), the descriptive norm (H4c) and the injunctive norm (H4d). Although this interaction has never been directly tested, the results are in line with past theoretical observations that social context influences individual attitudes and how normative information is perceived (Hogg & Smith, 2007; Livingstone & McCafferty, 2015). However, the interaction term did not successfully predict PBC (H4e). Only the main effects of group identification and the importance of drinking to the group identity were significant in predicting PBC. It seems that the higher one’s identification level, the lower their PBC over drinking. It is possible that someone who identifies highly with the group sees drinking as a social activity that involves less PBC, whereas someone who does not identify with the group could view drinking as an individualistic behavior. Similarly, as the importance of drinking to the group identity increases, one’s PBC over drinking decreases. It is possible that regardless of one’s level of identification, if the social group believe that drinking is important, there will be a perceived increase in social pressure to conform and engage in drinking.

Overall, the current study has been able to both replicate and extend the TPB. Whilst taking into account all the TPB variables simultaneously with advanced statistical analysis, the current study was able to confirm the findings of past research and concluded that attitudes, subjective norms and PBC predict binge drinking intentions, which in turn predict binge drinking behavior. The current study also helped to clarify the mixed evidence with regards to normative influence within the TPB. It appears beneficial to differentiate between the different types of social norms (subjective, descriptive and injunctive) as all three appear to have distinct effects on intentions. It is advised that when descriptive and injunctive norms are added into the TPB, they should not be measured as one construct, nor should they replace subjective norms, as the concepts are not interchangeable. Similarly, the results also demonstrate that measuring the descriptive and injunctive norms of the group is not the same as measuring the importance of the behavior to the group’s identity. It appears that one’s experience of the group and what it means to be a part of that group affects their perception of associated social norms.

Most notably, the current study demonstrates the SIA has much to contribute to understanding behavior in the context of the TPB. The present study revealed that it is not enough just to measure whether one identifies with a group or not when trying to predict behavior. It is beneficial to understand what behaviors are central to the salient group’s unique identity, in conjunction with how much an individual psychologically identifies with the group, as both will affect an individual’s perception of reality and ultimately their behavior. As four of the five interaction effects that were tested between group identification and the importance of the behavior to the group were significant predictors within the TPB, there is compelling evidence to argue that researchers could pay more attention to group processes when trying to predict one’s attitudes, PBC and perceptions of social norms in relation to drinking. Furthermore, being oriented to a situation as a group member may help explain the source of TPB constructs and variability across social contexts. In relation to binge drinking behavior, the TPB constructs can be viewed as outcomes of particular self-concepts becoming salient in a particular situation. Therefore, the current study has highlighted that a more comprehensive understanding of how the SIA can contribute to the TPB is highly valuable, as it advances our understanding of how to predict and potentially influence behavior.

### Limitations and Future Directions

Despite the current study’s important implications, there are some limitations that should be noted and rectified in future research. First, the current study only explores correlations between cross sectional variables, meaning that the causal direction of the relationships could be misinterpreted. For example, instead of one’s group membership predicting attitudes, it is possible that one’s attitudes will determine which group they chose to be a part of, as they seek out similar individuals (Kahler, Read, Wood, & Palfai, 2003). Similarly, instead of the importance of drinking to the group predicting perceived drinking norms, perceived drinking norms could influence whether one sees drinking as a behavior that is important. Therefore, future research needs to investigate the proposed relationships in both longitudinal and experimental designs to make stronger claims about the casual direction.

Second, some of the measurements used in the current study could be improved upon. Injunctive norms and PBC were only measured on two-item scales, which have been recognized as problematic (Marsh, Hau, Balla, & Grayson, 1998). Future studies could expand the number of items to capture these complex constructs in an attempt to increase content validity and measurement reliability (Eisinga, Te Grotenhuis, & Pelzer, 2013). The current study also measures descriptive norms, past behavior and drinking behavior in a way that is common within the social norm approach literature (for examples see Moore, Williams, Moore, & Murphy, 2013; Neighbors et al., 2010; Lewis & Neighbors, 2007), but not frequently utilized within the TPB. Although the measures have good test-retest reliability, good convergent validity (Marlatt et al., 1998; Neighbors et al., 2006) and are similar to common TPB measures, it is possible that this could affect the generalizability of the results. Additionally, using a variable centered perspective and turning descriptive norms, intentions and binge drinking behavior into binary variables could have resulted in losing valuable information. In future research, other ‘person-centered’ analysis options, such as latent profile analysis, might be able to preserve additional information.

Similarly, it is possible that when students are physically engaging in binge drinking within a social setting (such as a party or in a club), different and potentially conflicting social identities could be salient and affect behavior in a currently unknown way. It is necessary to see whether the current results can be replicated when other reference groups are made salient. Furthermore, as the current study was focused on specifically understanding binge drinking in university students, it would also be beneficial to replicate the proposed model with other health behaviors that range in social motivation (such as exercise, applying sunscreen, healthy eating etc.) and with other samples in the wider population, to further generalize the results.

In sum, the findings from the current study appear to offer promising extensions to the TPB when predicting binge drinking. It appears that viewing social identity processes as a background variable in the TPB offers insight into how the social environment can play a role in one’s drinking behavior. More specifically, group membership, including group identification and what behaviors are central to a group’s identity, seems to shape an individual’s perception of related attitudes, social norms and PBC. Therefore, perhaps a greater focus on social processes in line with the TPB should be encouraged in order to advance our ability to predict binge drinking behavior.
